# Experimental Evidence for Manure-Borne Bacteria Invasion in Soil During a Coalescent Event: Influence of the Antibiotic Sulfamethazine

**DOI:** 10.1007/s00248-022-02020-w

**Published:** 2022-05-12

**Authors:** Loren Billet, Stéphane Pesce, Fabrice Martin-Laurent, Marion Devers-Lamrani

**Affiliations:** 1 INRAE, UR RiverLy, Villeurbanne, France; 2 Agroécologie, INRAE, Institut Agro, Université de Bourgogne Franche-Comté, Dijon, France

**Keywords:** Community coalescence, Manure amendment, Sulfonamide, 16S rDNA sequencing, Firmicutes, Soil colonization

## Abstract

The fertilization of agricultural soil by organic amendment that may contain antibiotics, like manure, can transfer bacterial pathogens and antibiotic-resistant bacteria to soil communities. However, the invasion by manure-borne bacteria in amended soil remains poorly understood. We hypothesized that this kind of process is both influenced by the soil properties (and those of its microbial communities) and by the presence of contaminants such as antibiotics used in veterinary care. To test that, we performed a microcosm experiment in which four different soils were amended or not with manure at an agronomical dose and exposed or not to the antibiotic sulfamethazine (SMZ). After 1 month of incubation, the diversity, structure, and composition of bacterial communities of the soils were assessed by 16S rDNA sequencing. The invasion of manure-borne bacteria was still perceptible 1 month after the soil amendment. The results obtained with the soil already amended in situ with manure 6 months prior to the experiment suggest that some of the bacterial invaders were established in the community over the long term. Even if differences were observed between soils, the invasion was mainly attributable to some of the most abundant OTUs of manure (mainly Firmicutes). SMZ exposure had a limited influence on soil microorganisms but our results suggest that this kind of contaminant can enhance the invasion ability of some manure-borne invaders.

**Supplementary Information:**

The online version contains supplementary material available at 10.1007/s00248-022-02020-w.

## Introduction

Organic amendment enables fertilization of agricultural soils and allows the recycling of a substantial amount of waste produced by livestock such as manure [1]. This environmentally sound practice enhances crop yields by increasing the soil organic matter, phosphate, and nitrogen contents [2]. Besides these nutrient inputs, manure is also a source of exogenous biological material, mainly microorganisms, for amended soils [3]. This biological input may be helpful in carrying beneficial microorganisms for the functioning of agroecosystems [4] or even in suppressing plant and animal pathogens by antagonism [5, 6]. This biological input may be beneficial in carrying functionally interesting microorganisms [4] or even in suppressing plant and animal pathogens by antagonism [5]. However, it can introduce biological contaminants in soils and the rhizosphere of plants, such as pathogenic bacteria [7–9] or hazardous microbial functions such as antibiotic resistances [10, 11]. Therefore, understanding microbial outcomes of manure amendment in soils is of interest.

In this context, it is important to determine what enables or prevents a microbial invasion from manure to soils. In particular, knowledge is needed to better understand what make manure-borne microorganisms more likely to invade a soil, taking into consideration the influence of both the biotic and abiotic components of soil and manure (as well as their interactions). This question relates to the concept of microbial coalescence, which focuses on the outcome of mixing different environments, each presenting different biotic and abiotic characteristics [12, 13]. Amendment of agricultural soils with manure is an environmentally relevant example of coalescence event where the mix of two distinct environments results in a new one, namely an amended soil, with its own characteristics.

Previous studies showed that amended soils durably differ from manure-free soils when considering the abiotic part of coalescence [14, 15]. However, although the novel soil environment differs from the unamended one, the microbial community resulting from coalescence is generally relatively similar to that of the original one [16, 17]. The rather imbalanced ratio between soil and manure matrices may partially explain this. Moreover, it is also hypothesized that autochthonous soil microorganisms constitute a natural barrier preventing the establishment of allochthonous microorganisms originating from manure by outcompeting them [18, 19]. For instance, it has been shown that manure-borne bacteria that are adapted to, among other things, high nutrient content and anoxic conditions, tend not to be competitive with soil autochthonous bacteria [20].

Several environmental factors might influence the invasion success of manure-borne bacteria in soil. Firstly, based on the concept of coalescence, the invasion capacity is probably soil-dependent since it may vary according to the biotic (e.g., community structure and diversity) and abiotic (e.g., organic matter content) characteristics of the amended soil, which determine the potential presence of available ecological niches [21]. It can also be modulated by the repeated amendments, with two possible contradictory scenarios. On the one hand, transient and even unsuccessful invasion events can favor subsequent invasion [21]. On the other hand, the resistance of soil communities to invasion can be enhanced after a first invasion [22]. Another factor that can modulate invasion events in autochthonous microbiota refers to the possible presence of antibiotics in manure, which is frequently reported in the literature [23, 24]. These contaminants may disturb interaction patterns of microbial communities according to the antibiotic sensitivity profile of their respective members [25, 26], thus favoring the emergence of communities that would differ from those obtained under antibiotic-free environments. Since antibiotics are used on livestock [27], bacteria from animal tracts, which compose the manure community, may be more antibiotic-tolerant than non-antibiotic-exposed soil bacteria [28]. Indeed manure contains more antibiotic-resistant bacteria than soils [19, 29]. Consequently, contamination of soil with antibiotics originating from manure amendment is likely to promote the invading potential of antibiotic-resistant manure-borne bacteria in soils. In that sense, antibiotics could increase the invading potential of isolated antibiotic-resistant strains in soil [29, 30] and manure amendment can result in an increase of the diversity of antibiotic-resistant bacteria in soil [19].

In this context, the purpose of our microcosm study was to assess soil invasion by manure-borne bacterial OTUs during an agronomical coalescence event of soil fertilization with manure according to the presence or not of antibiotic pressure. We firstly hypothesized that the capacity of manure-borne bacteria to invade the amended soil would be variable according to the intrinsic abiotic and biotic properties of soils. In particular, among the four soils used, one was amended (in situ) 6 months earlier by the same manure to test whether previous amendment had an impact on invasion outcomes. In addition, we also hypothesized that some manure-origin OTUs would be distinguished from the emerging rare soil OTUs in response to the repetition of their invasive pattern in several of the tested soils. Finally, to verify whether antibiotics can interfere with the coalescence process by promoting the invasion of soils by manure-borne OTUs, a modality made of the antibiotic sulfamethazine (SMZ), which is commonly used in veterinary care, was considered [31, 32]. After 1 month of incubation, the diversity, structure, and composition of bacterial communities of the manure-amended soils exposed or not with SMZ were compared to those of their respective non-amended control soil and of the native manure by sequencing of 16S rDNA.

## Material and Method

### Collection and Analyses of Soils and Manure

The four soils, named A, B, C, and D, are anthrosols selected for their different textures and physico-chemical characteristics (see Supplementary table 1 for details). They were sampled from the upper 20 cm of different agricultural fields the Bourgogne–Franche–Comté region of France in November 2018. They originated respectively from an agricultural field frequently flooded by the nearby Saône River, the same plot but at a higher altitude, the experimental farm of Epoisses, and a field near a hog nursery that had received manure 6 months before the sampling. Each soil was air-dried to 60% of their water content at pF 2.7, sieved (mesh size 5 mm), and stored about 2 months in airtight plastic bags at 4°C until use. The manure was collected from the pig nursery whose manure had been previously used to fertilize soil D.

### Microcosm Experiment Setup

The experiment consisted of four different soils amended or not with manure and treated or not with SMZ. Considering four conditions and five replicates per condition, 20 microcosms for each soil were prepared as described below. Fifty grams dry weight (DW) of soils were placed in glass bottles closed with air-permeable lids made of gauze and cotton. In order to gently activate the native soil microorganisms, soil microcosms humidified up to 80% of their water content at pF 2.7, were incubated for 1 week at 20°C (±1°C). SMZ sodium salt [SMZ, CAS number: 1981-58-4, 4-amino-*N*-(4,6-dimethyl-2-pyrimidinyl)benzenesulfonamide, Sigma-Aldrich, France] was diluted to 100 mg ml^−1^ in water and filtered (0.2 μm). Before applying treatments, the manure was incubated overnight at room temperature (20 ± 1°C). Treatments were prepared a few minutes before their application on soils to reach nominal concentrations of 100 mg of SMZ kg^−1^ dry soil and 13 ml of manure kg^−1^ dry soil. Then, the microcosms were incubated for 1 month in the dark at 20°C (±1°C). Their humidity has been kept stable by regular watering.

### Soil DNA Extraction and 16S rDNA Sequencing

DNA was extracted from subsamples of 250 mg of soil for each microcosm using the DNeasy PowerSoil-htp 96-well DNA isolation kit (Qiagen, France) following the manufacturer’s instructions and stored at −20°C. As previously described by Billet et al. (2021), the V3–V4 hypervariable region of the bacterial 16S rRNA gene was amplified using a two-step PCR and sequenced on MiSeq (Illumina, 2 × 250 bp) and a Jupyter Notebook developed in-house was used to analyze the sequence data [30]. Briefly, sequences were assembled using PEAR [33] with default settings. Further quality checks were conducted using the QIIME pipeline [34] and short sequences were removed (<400 bp). Reference-based and de novo chimera detection together with clustering in OTUs were performed using VSEARCH [35] and Greengenes’ representative set of 16S rRNA sequences as the reference database. The identity thresholds were set at 94%. Representative sequences for each OTU were aligned using Infernal [36] and a 16S rRNA phylogenetic tree was constructed using FastTree [37]. Taxonomy was assigned using RDP Classifier [38] and the latest released Greengenes database (v.05/2013; [39]). α-Diversity metrics (PD whole tree) and UniFrac distance matrices [40] were determined from rarefied OTU tables of 25,700, 24,500, 28,300, and 23,100 sequences per sample for soils A, B, C, and D, respectively. Sequences were deposited in the SRA at NCBI under the accession number SUB8712901.

### Statistical Analyses

Statistical analyses were carried out using RStudio statistical software (version 1.2.5033). For each soil, we used analysis of variance (ANOVA) model to determine the effects of treatments on the alpha diversity indices of bacterial communities. Normality and homogeneity of the residual distribution were inspected and log10-transformations were performed when necessary.

Permutational multivariate analysis of variance (PermANOVA) was used to test significant differences in communities’ structure using adonis function implemented in the vegan package (permutations = 999).

For each soil, the OTU table was filtered (relative abundance >0.05%) and to detect the OTUs significantly impacted by treatments (antibiotic treatment and manure amendment) a generalized linear mixed model was developed. Considering that, the OTU abundance *Y* follows a Poisson law of parameter Λ as $\mathrm{Y}\sim \mathcal{P}\left(\varLambda \right)$, we used the following model :

$$\log \left({\Lambda}_{ij k}\right)={o}_{ij}+\upmu +{\upalpha}_i+{\beta}_j+{\left( a\beta \right)}_{ij}+{I}_k+{Z}_{ij k},\kern0.5em \mathrm{iid}\sim \mathcal{N}\left(0,{\sigma}^2\right)$$

*o*_*ik*_ is the offset calculated as the log of the sample read sum. μ is the intercept. α_*i*_ is the fixed effect of manure amendment (*i* = 1,2). *β*_*j*_ is the effect of SMZ treatment (*j* = 1,2). (*aβ*)_*ij*_ is the interaction effect between the manure amendment and SMZ treatment. *I*_*k*_ is the random effect of the individuals. *Z*_*ijk*_ is the residual error.

The analysis was performed using the glmer function of the lme4 package (version 1.1-27). Chi-squared test was performed to detect significant pairwise differences for each treatment (significance threshold set at *p* < 0.05). Subsequently, we performed a post hoc Tukey test with the emmeans function of the emmeans package (version 1.6.1) to detect significant differences caused by manure amendment depending of treatment with SMZ (significance threshold was set at *p* < 0.05). A Bonferroni correction was used to decrease substantially the probability of detecting false positives (significance threshold was set at 0.05).

For the specific case where OTUs had a null abundance in soil non-amended with manure, we added a specific filter: for each OTU, systematic detection in every replicate of condition with manure was considered significant. Furthermore, only OTUs with a size effect implying the doubling of their abundance between treatments were retained.

## Results

### Manure’s Effect on Diversity in the Soil Communities

**Fig. 1:**
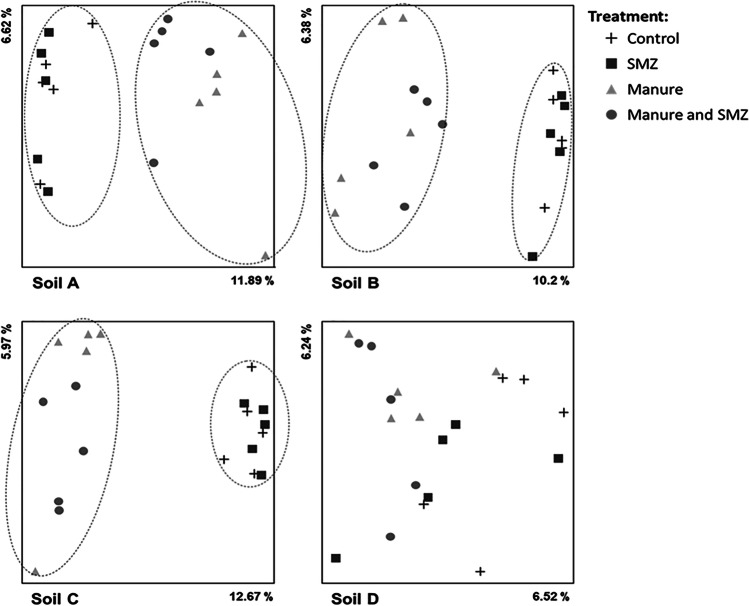
Comparison of the bacterial diversity in the four soils (A, B, C, and D) amended or not with manure and exposed of not to SMZ. Principal coordinates analysis (PCoA) of the unweighted Unifrac distance matrices of 16S rDNA amplicon sequences showing changes in bacterial community structure. The first two axes and the percent of variation explained by each are indicated. Significant effects of manure amendment are represented by ellipse (*p*<0.001)

**Fig. 2:**
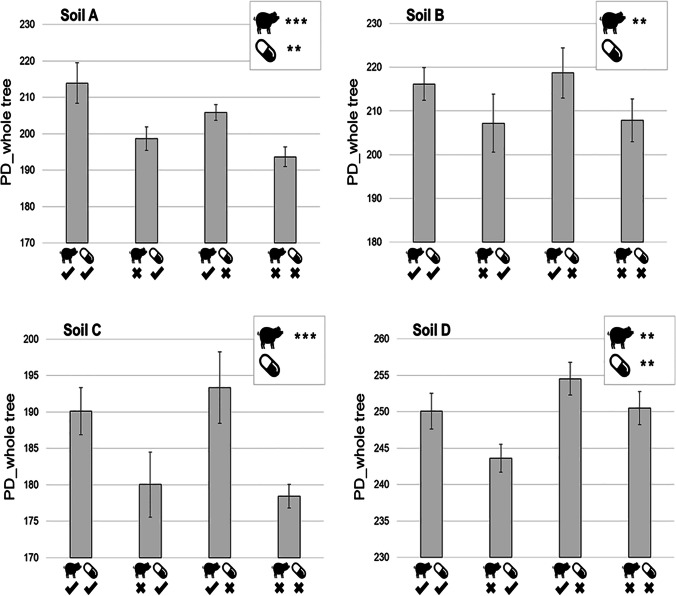
Phylogenetic diversity of the bacterial diversity in the four soils (A, B, C, and D) amended or not with manure and exposed or not to SMZ. The PD whole tree index of the four soils either amended or not with manure (pig symbol) and exposed or not with SMZ (pills symbol) are represented. Bar indicated the standard deviation of the mean (*n* = 5). For each soil, a statistical impact of the manure amendment or of the exposure to SMZ on the calculated index is indicated (ANOVA, *p < 0.05, **p < 0.01, ***p < 0.001)

Principal component analysis (PCoA) performed on unweighted unifrac matrices showed that after 1 month, the composition of the bacterial community in soils A, B, and C amended with manure was distinct from those not amended (Fig. 1, PERMANOVA, *p*<0.001). Only the community structure of soil D, which received in situ manure from the same pig nursery 6 months before the experiment, was not impacted by manure amending. Therefore, after the 1-month incubation period, the structure of bacterial communities in three of the four soils differed depending on manure amendment modality. Regarding alpha diversity, manure amendment significantly increased the PD whole tree index in all the soils, including soil D (Fig. 2, ANOVA, *p*<0.001 for soils A and C, *p*<0.01 for soils B and D). This indicates that phylogenetic diversity of bacterial community was higher in soils amended with manure than in those not amended.

### Manure’s Effect on the Relative Abundance of OTUs

**Fig. 3:**
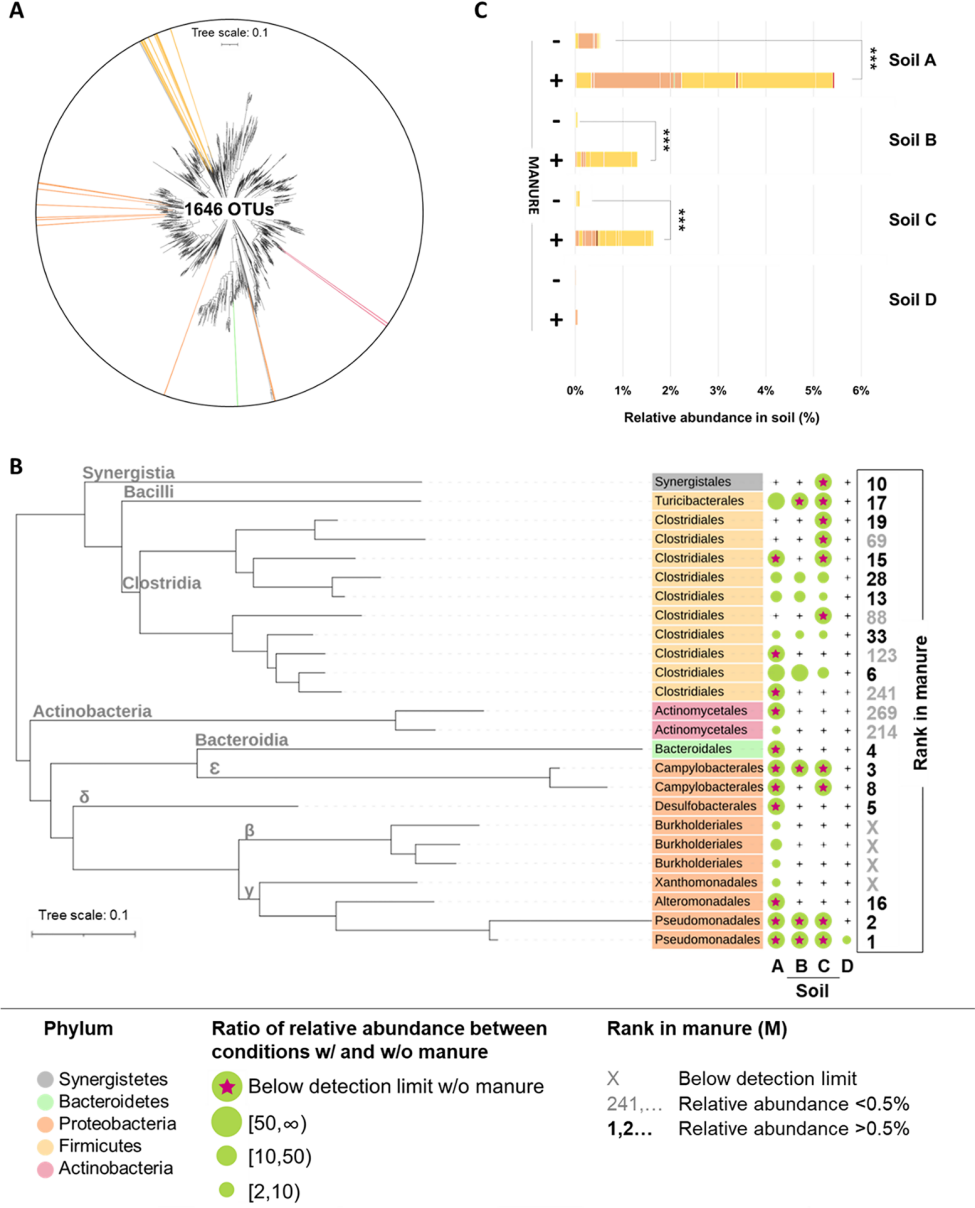
Impact of soil amendment with manure on the relative abundance of bacterial OTUs. (a) Phylogenetic tree representing all the studied OTUs (relative abundance > 0.05% in at least one soil). Among them the ones associated with a colored line were the ones that were significantly impacted by manure amendment. (b) For each of the four soils impact of amendment with manure on the relative abundance of significantly impacted OTUs. Colors of leaves indicate the phylum of the OTU. The strength of the impact is indicated by the size of green circle which corresponds to the ratio of the relative abundance of the OTU between condition with and without manure. The symbol “+” indicates that the manure does not have a significant impact on the corresponding OTU in this soil. For each OTU, its relative abundance in the manure is indicated as a rank. (c) Abundance relative of OTUs impacted by manure amendment in soils amended (+) or not (−) with manure. Colors are phylum indication. Each segment corresponds to an OTU

The effect of manure on the structure of communities observed by PCoA analysis relied on few OTUs (Fig. 3). Indeed, among the 1646 OTUs with relative abundance > 0.05% in at least one soil, only 25 OTUs were significantly impacted by amendment with manure (Fig. 3a, ANOVA, *p*<0.05): 10 OTUs related to Proteobacteria, 11 to Firmicutes, 2 to Actinobacteria, 1 to Synergitetes, and 1 to Bacteroidetes (Fig. 3b). For each soil, significant effects of amendment with manure were observed on 20 OTUs (among 642) in soil A, 9 OTUs (among 639) in soils B, and 14 OTUs (among 671) in soil C. In soil D, only 1 of the 826 OTU was significantly affected by manure.

Soil amendment with manure positively influenced the relative abundance of all these OTUs (Fig. 3b). Some of them were only detected in conditions with manure (11 in soil A, 5 in soil B, and 10 in soil C, see red stars in Fig. 3b), while others increased by a factor of 2.6 to 262 in amended soils (see size of green circles in Fig. 3b). Altogether, the manure-induced increase in the relative abundance of each impacted OTUs resulted in a noticeable increase in the sum of these relative abundances at the community level going up from 0.5% to 5.4%, 0.1% to 1.3%, and 0.1% to 1.6% in soil A, B, and C, respectively (Fig. 3c). In contrast, the effect of manure on only 1 OTU in soil D had almost no effect at community level (Fig. 3c), as observed previously with the PCoA analysis (Fig. 1).

Among the 25 different OTUs impacted by amendment with manure in at least one soils, 21 were abundantly detected in manure, with 15 of them even being among the 35 most abundant OTUs in manure where their relative abundance was greater than 0.5% (manure diversity = 654 OTUs). Interestingly, the comparison of the four manure-amended soils showed that 10 of these 15 OTUs had a redundant pattern in at least two of them. Among them, 1 Pseudomonodales was significantly impacted by manure in all four soils without exception. It was the most abundant in manure explaining by itself 11.4% of its composition while it was only detected in soil D and not in soil A, B, and C under manure-free conditions. Seven other OTUs (4 Clostridiales, 1 Turibacterales, 1 Campylobacterales, 1 Pseudomonodales) were significantly impacted in all four soils except in soil D. It is noteworthy that these OTUs were always more abundant in soil D under manure-free conditions than in others (Supplementary Tab 2). In addition, 1 Clostridiales and 1 Campylobacter were not detected under manure-free conditions in both soils A and C.

### Influence of SMZ on Manure’s Effect

**Fig. 4:**
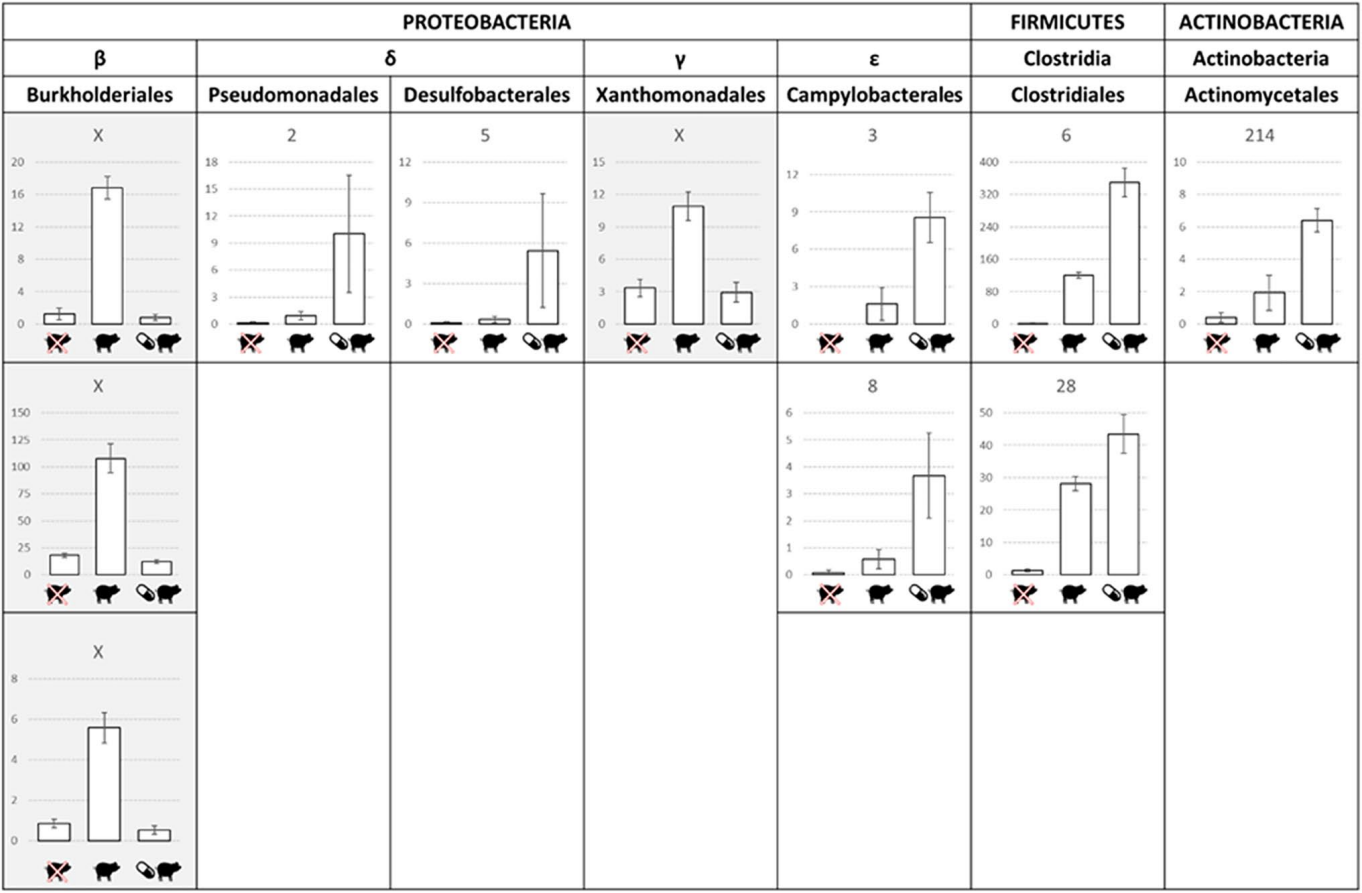
Abundance of OTUs impacted differently by manure amendment depending on SMZ application. The abundance of the OTU in absence of manure whether or not exposed to SMZ, in presence of manure and in presence of manure and SMZ are indicated above respectively the “Strikethrough pig”, “pig” and “pill and pig” symbols. Bars indicated the standard deviation of the means (*n* = 5). Each OTU is entitled by its rank in the manure

SMZ treatment had a significant impact on bacterial community structure only for the soil A when considering the weighted unifrac matrix (Supplementary Fig 1, PermANOVA, *p*<0.001). This indicates that in the soil A SMZ did not significantly affect the presence and absence of OTUs but only the relative abundance of some of them. Eleven OTUs responded to manure spreading depending on SMZ (Fig. 4, *p*<0.05). They all were related to the 25 OTUs whose abundance was increased by manure. Two response patterns were observed. For 4 OTUs not detected in manure, SMZ exposure inhibited the stimulating effect of manure as compared to soil not exposed to SMZ. For the other 7 OTUs, SMZ exposure enhanced the increasing effect of manure on their abundance in a non-negligible way. Indeed, the sum of their relative abundance increased from 2.4% to 4.5% with SMZ addition (data not shown). For this latter pattern, it corresponded to OTUs always detected in manure, one being even only detected in soil amended with manure.

## Discussion

The influence of manure amendment on the soil bacterial communities was still noticeable after 1 month, although the magnitude of changes varied between soils. The bacterial community of soil A showed, compared to others, a stronger shift following the manure amendment, while that of soil D was the least affected. The most abundant OTUs in the soils remained stable but the rare ones were affected (i.e., the rarest one among those with relative abundance > 0.05%) and possibly undetected ones (i.e., those with relative abundance < 0.05% and/or those that were not present in the soil). Changes in the composition of microbial communities due to rare OTUs during coalescence processes are in line with the observation of the emergence of the rare-biosphere microorganisms following environmental disturbances [41]. The least abundant OTUs under stable conditions are often those favored by a disruption of the environmental conditions [41, 42]. They may benefit from the equilibrium disruption of the environment where they are normally outcompeted by the better-adapted to multiply and colonize newly available ecological niches [43].

The communities of soils amended with manure exhibited OTUs with a wider distribution along the phylogenetic tree. This increase in diversity within the soil bacterial community could either correspond to the emergence of endogenous rare OTUs with functional abilities better adapted to this new environment than the original one or to the invasion of soil community. While previous studies provided evidence suggesting soil invasion by manure-borne bacteria, they could not distinguish surely allochthonous invaders from autochthonous OTUs that emerge because of environmental disturbance caused by the adjunction of exogenous matter [17, 22]. In the same line, we showed that some OTUs favored in response to manure amendment were not detected in the manure-free soils although they were almost all detected in the manure. However, even if these OTUs were not detected in control soils but detected in the manure, it is not possible to conclude definitively that there was an effective invasion of the soil with OTUs of manure origin since the lack of detection in the soil could be due to sequencing biases. An absence of detection by 16S rDNA sequencing does not necessarily mean its effective absence in the community because of detection thresholds that ignore the endogenous rarest OTUs and make it impossible to perceive the entire bacterial community [44].

However, through the use of different soils, including one already amended in situ with pig manure 6 months before its sampling (i.e., soil D), we provided additional evidences supporting the hypothesis of invasion by manure-borne OTUs. First, in response to manure amendment, some of these OTUs presented the same increase pattern in different soils. The extreme case involved the most abundant OTU in the manure, a Pseudomonodales, which was favored in each of the four soils and was not detected in control soils A, B, and C not amended with manure. This response pattern suggests a common manure origin since all the soils presented different biotic and abiotic properties, limiting the chance that they hosted the same rarest OTUs. Secondly, all these OTUs were detected in the non-experimentally amended soil D, suggesting that they were successful in colonizing this soil following previous in situ manure amendment events (the last amendment having taken place 6 months before the experiment).

Considering this set of arguments, we assumed that all or almost all the OTUs detected in both manure and soil D and which increased in several soils were from manure origin. All of them were among the most abundant OTUs in manure. Their high abundance in manure bacterial community composition may explain the success of their invasion, since a high inoculation rate is more likely to be successful [22, 45]. One could argue that the detection of the most abundant bacteria of the manure in soil may be a bias due to DNA from dead cells that did not establish in the soil [46, 47]. However, this argument can be rejected since not all the most abundant OTUs from manure were detected in the amended soils.

Interestingly, when examining the abundance of the OTUs that are suspected to be invaders established in soil D during previous in situ amendment of manure (see above), two kinds of establishment patterns can be differentiated, depending on their taxonomic affiliation: the first one relates to the Firmicutes that are quite abundant in soil D, while the other relates to the Proteobacteria that are rare OTUs (Supplementary Tab.2). The ability of OTUs related to Firmicutes to have become well represented in the soil following previous manure amendment may be because of their physiological attributes. Firmicutes, contrary to Proteobacteria, have sporulation capacity that may confer upon them an ability to persist for a long period in a less hospitable environment until favorable conditions return [48]. The long-term survival of Firmicutes in soil after manure amendment has been already reported, with detection even after the winter season [17].

It is noticeable that the community of soil D was much less impacted by manure amendment than the other soils, with only one OTU being significantly increased. This is in contrast to the hypothesis formulated by Mallon et al. (2018), who suggested that previous invasions generate legacy effects in soil communities facilitating future invasion attempts [21]. However, it is consistent with the study of Gravuer and Scow, (2021), who observed that soils’ communities after a first disturbance by manure amendment were less impacted by the following ones [22]. They hypothesized that the endogenous soil bacteria may adapt to the occasional input of nutrients carried by manure, outcompeting manure-borne bacteria during subsequent amendments. Here, we rather hypothesized that it is the manure-borne OTUs already established in the soil that prevent the new ones from establishing since they are functionally similar and already adapted to soil.

Finally, the impact of the antibiotic SMZ on the structure of autochthonous soil bacterial communities was relatively low as it was significant in only one of the four soils (soil A). Previous results obtained from the same microcosms may explain this fact [30]. After 1 month of incubation, the bioavailable fraction of SMZ in the SMZ-treated soils represented a low level of exposure for the community. Concordantly, the soil A in which the free-fraction of SMZ was the highest [30] is the one for which its bacterial community was differently impacted by manure amendment according the presence or not of SMZ. A first group of seven OTUs, most of them suspected of being manure-borne OTUs, had their abundance even more increased by manure amendment in the presence of SMZ. The observed effect of SMZ could be explained by the selection of antibiotic-resistant bacteria. Persistence of resistant bacteria and contamination by antibiotics could be positively linked [29]. For instance, we showed in a previous study that the invasion of SMZ-resistant bacteria in soil can be favored in the presence of the antibiotic [30]. The second group consisted of OTUs that were favored by the manure amendment in the absence of SMZ but that lost this advantage in the presence of the antibiotic. All were autochthonous soil bacteria. The lack of negative effects of SMZ on these OTUs in manure-free conditions (Supplementary Tab 2) suggests that they were not especially sensitive to SMZ.

## Conclusion

Manure-borne OTUs can colonize soils during the coalescence event of manure amendment. Changes in the composition of bacterial community were mainly attributable to the most abundant OTUs of manure (mainly Firmicutes) with some variations due to soil type. At times, their establishment in bacterial community of soils was still perceptible 1 month after manure amendment. The idea of their long-term establishment was reinforced by their detection in the soil that was amended in situ 6 months prior its sampling for this experiment. SMZ treatment had a limited influence on soil bacterial composition, but in one of the four soils, it enhanced the invasion potential of some manure-borne invaders. It would be interesting to clarify their antibiotic resistant ability.

## Supplementary Information

Supplementary file1 Supplementary Fig 1 Comparison of the bacterial diversity in the four soils (A, B, C and D) amended or not with manure and exposed or not to SMZ. Principal coordinates analysis (PCoA) of the weighted Unifrac distance matrices of 16S rDNA amplicon sequences showing changes in bacterial community structure. The first two axes and the percent of variation explained by each are indicated. Significant effects of amendment of manure and exposure to SMZ are represented by ellipse (P<0.001). (PNG 234 kb)

High resolution image (TIF 10023 kb)

Supplementary file2 Supplementary Tab 1 GPS coordinates and physical-chemical properties of the four different soils used in the study. (PDF 128 kb)

Supplementary file3 Supplementary Tab 2 Phylogeny and relative abundance of the 10 OTUs that were significantly increased in several soils amended with manure (expressed per 10 000 OTUs counted). (PDF 282 kb)

## Data Availability

The datasets generated during the current study are available from the corresponding author on reasonable request.
