# Supplementary figures and images for: Experimental Evidence for Manure-Borne Bacteria Invasion in Soil During a Coalescent Event: Influence of the Antibiotic Sulfamethazine
Source: Microb Ecol. 2022 May 12;85(4):1463–72. doi: 10.1007/s00248-022-02020-w (PMC10167166; doi:10.1007/s00248-022-02020-w)

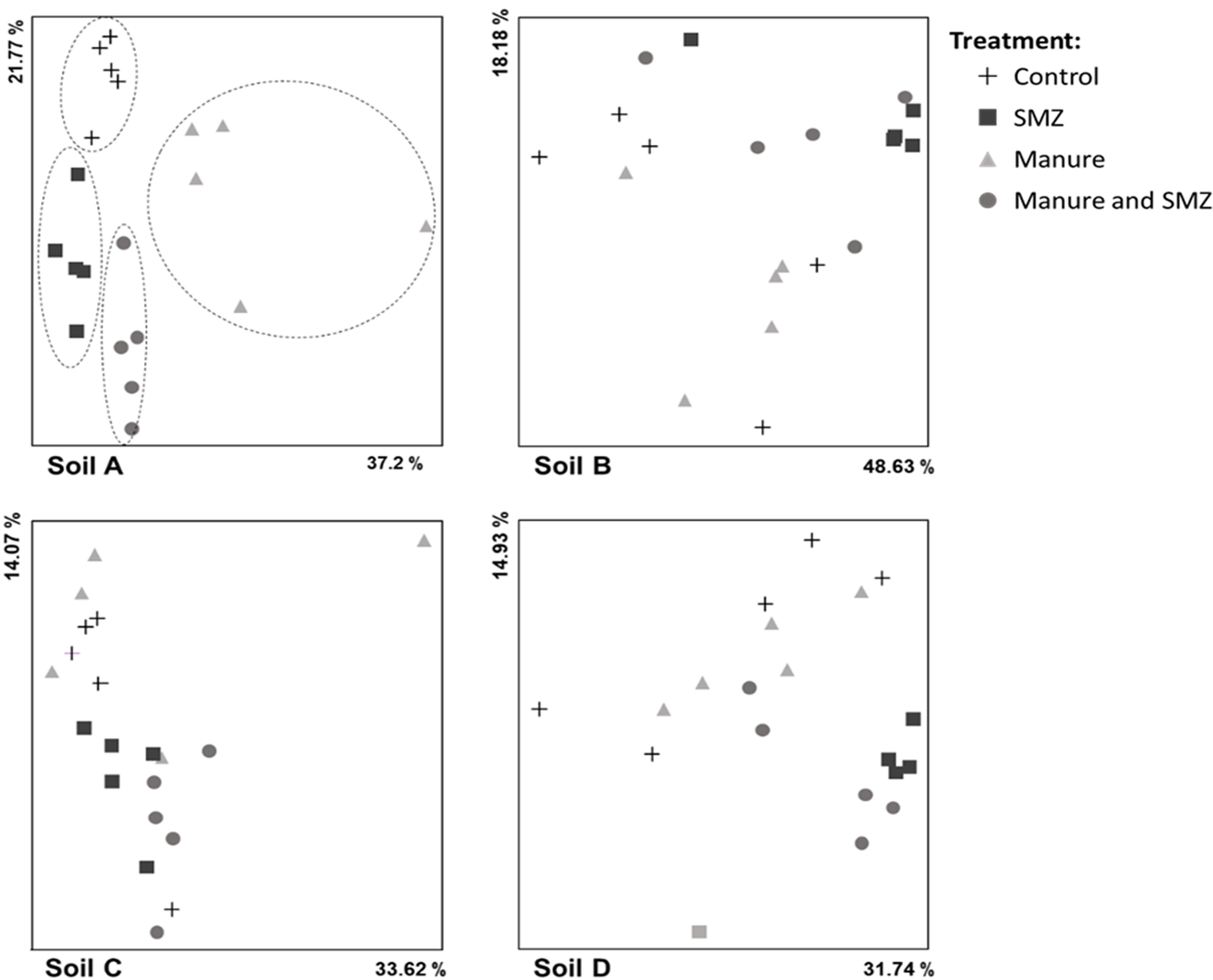

Supplement: Supplementary file 1 — Supplementary file1 Supplementary Fig 1 Comparison of the bacterial diversity in the four soils (A, B, C and D) amended or not with manure and exposed or not to SMZ. Principal coordinates analysis (PCoA) of the weighted Unifrac distance matrices of 16S rDNA amplicon sequences showing changes in bacterial community structure. The first two axes and the percent of variation explained by each are indicated. Significant effects of amendment of manure and exposure to SMZ are represented by ellipse (P<0.001). (PNG 234 kb) [file 248_2022_2020_Fig5_ESM.png]

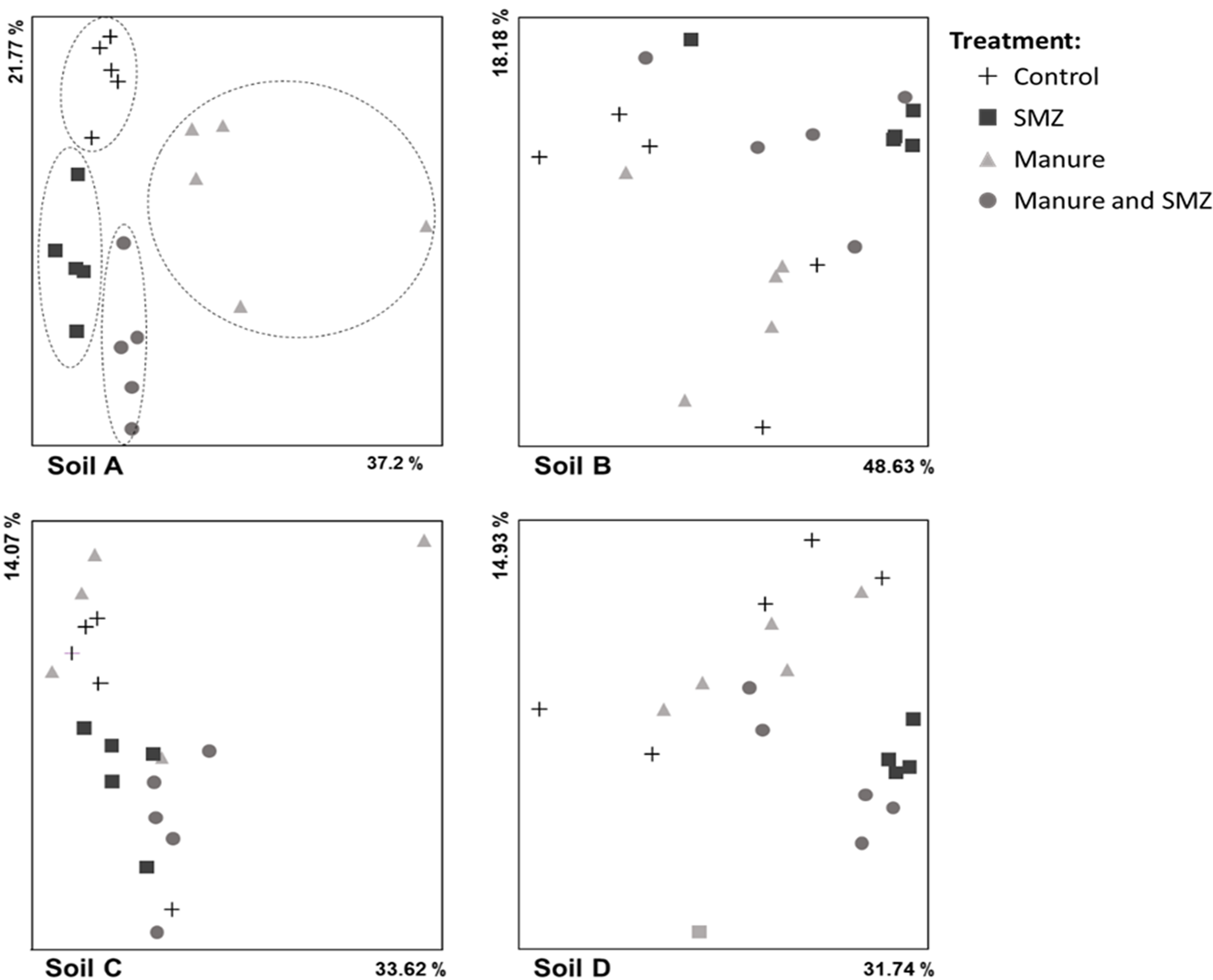

Supplement: Supplementary file 2 — High resolution image (TIF 10023 kb) [file 248_2022_2020_MOESM1_ESM.tif]
