# Supplementary material for: Experimental Evidence for Manure-Borne Bacteria Invasion in Soil During a Coalescent Event: Influence of the Antibiotic Sulfamethazine
Source: Microb Ecol. 2022 May 12;85(4):1463–72. doi: 10.1007/s00248-022-02020-w (PMC10167166; doi:10.1007/s00248-022-02020-w)
Supplement: Supplementary file 3 — Supplementary file2 Supplementary Tab 1 GPS coordinates and physical-chemical properties of the four different soils used in the study. (PDF 128 kb) [file 248_2022_2020_MOESM2_ESM.pdf]

		Soil A	Soil B	Soil C	Soil D
**GPS COORDINATES**					
Latitude	-	47.239562	47.239506	47.231351	46.960258
Longitude	-	5.415079	5.416419	5.104477	5.483326
**PHYSICAL-CHEMICAL PROPERTIES**					
Clay	‰	84	215	360	146
Fine silt	‰	37	111	338	332
Coarse silt	‰	32	52	238	166
Fine sand	‰	117	147	37	64
Coarse sand	‰	731	474	28	292
pH	-	5.6	6.1	6.9	6.1
Total carbonates	‰	0	0	0	0
Total organic carbon	‰	5.2	9.9	13	10.4
Organic matter	‰	9	17.1	22.4	18
Total nitrogen	‰	0.54	1.15	1.37	1.26
C/N ratio	-	9.6	8.6	9.5	8.2
Metson CEC	Me/kg	41	112	165	79
Water content at pF 2.7	%	6.8	14	21.4	18.3
Water content at saturation	%	28.5	37.1	39.1	40.8
SMZ concentration	µg/kg	0.63	0.43	< 0.08	< 0.08
