# Supplementary material for: Experimental Evidence for Manure-Borne Bacteria Invasion in Soil During a Coalescent Event: Influence of the Antibiotic Sulfamethazine
Source: Microb Ecol. 2022 May 12;85(4):1463–72. doi: 10.1007/s00248-022-02020-w (PMC10167166; doi:10.1007/s00248-022-02020-w)
Supplement: Supplementary file 4 — Supplementary file3 Supplementary Tab 2 Phylogeny and relative abundance of the 10 OTUs that were significantly increased in several soils amended with manure (expressed per 10 000 OTUs counted). (PDF 282 kb) [file 248_2022_2020_MOESM3_ESM.pdf]

		Relative abundance *(per 10 000 OTU)*				Phylogeny		
**Rank in manure**	**Soil impacted**	**Soil A**	**Soil B**	**Soil C**	**Soil D**	**phylum**	**class**	**order**
8	A/C	BDL	BDL	BDL	**0.1 ± 0.1**	Proteobacteria	ε	Campylobacterales
15	A/C	0.1 ± 0.1	BDL	BDL	**0.3 ± 0.2**	Firmicutes	Clostridia	Clostridiales
3	A/B/C	BDL	0.1 ± 0.1	BDL	**0.3 ± 0.4**	Proteobacteria	ε	Campylobacterales
2	A/B/C	0.1 ± 0.1	0.1 ± 0.1	BDL	**0.5 ± 0.4**		γ	Pseudomonadales
1	A/B/C/D	0.1 ± 0.2	0.1 ± 0.2	BDL	**0.9 ± 0.6**			
17	A/B/C	0.2 ± 0.2	0.1 ± 0.1	0.2 ± 0.3	**29.6 ± 4.2**	Firmicutes	Bacilli	Turicibacterales
28	A/B/C	1.3 ± 0.5	0.3 ± 0.2	0.8 ± 0.9	**39.4 ± 7.9**		Clostridia	Clostridiales
33	A/B/C	5.0 ± 1.3	1.1 ± 0.6	1.1 ± 1.4	**60.0 ± 6.7**			
13	A/B/C	2.2 ± 1.0	2.7 ± 0.8	2.6 ± 2.6	**87.9 ± 13.3**			
6	A/B/C	1.3 ± 0.6	0.3 ± 0.2	3.7 ± 5.4	**211.5 ± 23.2**			
